# Supplementary material for: Acid Sphingomyelinase Deficiency Type B Patient-Derived Liver Organoids Reveals Altered Lysosomal Gene Expression and Lipid Homeostasis
Source: Int J Mol Sci. 2023 Aug 10;24(16):12645. doi: 10.3390/ijms241612645 (PMC10454326; doi:10.3390/ijms241612645)
Supplement: Supplementary file 1 [file ijms-24-12645-s001.zip › ijms-2517716-supplementary.pdf]

Supplementary material

1. **Table S1:** Total amount of lipids measured in liver organoids from control and ASMD type B. Lipid species are shown in order from most to least abundant.*SD: Standard deviation; ** P-value is referred to comparison with control organoids.

Lipid Class		Control		ASMD type B		
		Average (pmol)	SD*	Average (pmol)	SD*	p-value**
Cholesterol	Chol	5070.61	488.56	4592.23	503.14	0.175
Triacylglycerol	TAG	776.33	40.78	1544.77	175.92	0.048
Phosphatidylethanolamine	PE	1750.48	169.52	947.04	84.90	0.042
Phosphatidylcholine	PC	1151.01	98.63	693.33	46.36	0.007
Phosphatidylinositol	PI	361.83	17.42	337.68	26.10	0.469
Phosphatidylethanolamine (-ether)	PE O-	176.58	16.26	240.80	21.14	0.100
Sphingomyelin	SM	141.20	15.16	227.21	24.73	0.039
Ceramide	Cer	148.94	11.99	197.44	17.10	0.049
Phosphatidylserine	PS	273.10	32.14	170.92	19.05	0.060
Cholesterol esters	CE	13.47	0.59	160.43	17.96	0.014
Diacylglycerol	DAG	97.13	6.39	55.85	4.49	0.015
Phosphatidylglycerol	PG	18.41	1.87	49.39	5.20	0.021
Hexosylceramide	HexCer	62.00	6.41	33.46	2.94	0.058
Phosphatidylcholine (-ether)	PC O-	12.18	0.39	30.90	2.50	0.008
Lyso-Phosphatidylcholine	LPC	22.38	2.17	26.01	3.52	0.224
Phosphatidate	PA	22.70	1.14	17.19	2.07	0.079
Cardiolipin	CL	16.56	0.37	13.19	0.82	0.033
Lyso-Phosphatidylethanolamine (-ether)	LPE O-	2.32	0.22	5.05	0.48	0.034
Lyso-Phosphatidylserine	LPS	3.12	0.53	4.57	0.46	0.235
Lyso-Phosphatidylinositol	LPI	2.70	0.41	3.27	0.40	0.275
Lyso-Phosphatidylcholine (-ether)	LPC O-	1.12	0.18	2.73	0.29	0.057
Lyso-phosphatidylglycerol	LPG	1.24	0.09	2.01	0.11	0.023
Lyso-Phosphatidylethanolamine	LPE	1.39	0.14	1.00	0.08	0.131
Lyso-Phosphatidate	LPA	0.96	0.08	0.46	0.05	0.037

Table S2: List of the top lysosome related DEGs down-regulated after differentiation of liver organoids.

GENE_ID	NAME_GENE	Average Expression	log2 (fold change)[1]	pvalue[2]	padj[3]
ATP6V0D1	ATPase H+ transporting V0 subunit d1	1865.15	-0.37	0.0001	0.0008
CD63	CD63 molecule	12104.37	-0.34	0.0000	0.0001
DMXL2	Dmx like 2	233.17	-0.87	0.0000	0.0000
AP1G2	adaptor related protein complex 1 subunit gamma 2	1121.80	-0.63	0.0000	0.0000
AP3B1	adaptor related protein complex 3 subunit beta 1	642.25	-1.07	0.0000	0.0000
FUCA1	alpha-L-fucosidase 1	2368.32	-0.69	0.0000	0.0000
ARSA	arylsulfatase A	940.58	-0.44	0.0004	0.0018
CTSA	cathepsin A	3494.65	-0.25	0.0006	0.0028
CTSE	cathepsin E	41030.68	-0.75	0.0000	0.0000
CTSF	cathepsin F	248.85	-0.75	0.0003	0.0017
CTSH	cathepsin H	2693.53	-0.53	0.0000	0.0000
CTSV	cathepsin V	425.42	-3.18	0.0000	0.0000
CTSZ	cathepsin Z	4617.22	-0.59	0.0000	0.0000
CTNS	cystinosin, lysosomal cystine transporter	144.08	-1.20	0.0006	0.0027
ENTPD4	ectonucleoside triphosphate diphosphohydrolase 4	1059.13	-0.50	0.0004	0.0019
GALNS	galactosamine (N-acetyl)-6-sulfatase	615.78	-0.80	0.0000	0.0000
GM2A	ganglioside GM2 activator	630.12	-0.35	0.0012	0.0053
HEXB	hexosaminidase subunit beta	1446.14	-0.75	0.0000	0.0000
HYAL1	hyaluronidase 1	5204.75	-1.38	0.0000	0.0000
HYAL2	hyaluronidase 2	1457.98	-0.38	0.0003	0.0017
LIPA	lipase A, lysosomal acid type	637.63	-0.89	0.0000	0.0000
PSAP	prosaposin	22061.46	-0.26	0.0003	0.0017
SMPD1	sphingomyelin phosphodiesterase 1	2260.37	-0.93	0.0000	0.0000
SUMF1	sulfatase modifying factor 1	1073.01	-0.76	0.0000	0.0000

[1]**log2FoldChange:** log2 fold change between the groups; [2]**pvalue:** Wald test p-value; [3]**padj:** Benjamini-Hochberg adjusted p-value.

Table S3: List of the top lysosome related DEGs up-regulated after differentiation of liver organoids.

GENE_ID	NAME_GENE	Average Expression	log2 (fold change)[1]	pvalue[2]	padj[3]
ATP6V0B	ATPase H+ transporting V0 subunit b	1473.99	0.54	0.0000	0.0000
CD68	CD68 molecule	3694.96	1.44	0.0000	0.0000
GNPTAB	N-acetylglucosamine-1-phosphate transferase subunits alpha and beta	333.86	0.56	0.0018	0.0073
ASAH1	N-acylsphingosine amidohydrolase 1	1421.49	0.49	0.0000	0.0000
NPC1	NPC intracellular cholesterol transporter 1	723.62	0.58	0.0000	0.0000
TCIRG1	T cell immune regulator 1, ATPase H+ transporting V0 subunit a3	2229.93	0.91	0.0000	0.0000
ACP5	acid phosphatase 5, tartrate resistant	198.51	4.05	0.0000	0.0000
GAA	alpha glucosidase	1702.46	0.71	0.0000	0.0000
IDUA	alpha-L-iduronidase	251.44	0.72	0.0002	0.0010
NAGA	alpha-N-acetylgalactosaminidase	558.86	0.91	0.0000	0.0000
CTSB	cathepsin B	7175.67	1.22	0.0000	0.0000
CTSD	cathepsin D	40423.33	0.60	0.0000	0.0000
CTSL	cathepsin L	750.30	1.38	0.0000	0.0000
CTSS	cathepsin S	1140.01	0.49	0.0000	0.0000
DNASE2	deoxyribonuclease 2, lysosomal	647.52	0.59	0.0000	0.0000
GUSB	glucuronidase beta	1161.03	0.74	0.0000	0.0000
IDS	iduronate 2-sulfatase	783.31	0.94	0.0000	0.0000
LAMP2	lysosomal associated membrane protein 2	2333.75	0.31	0.0002	0.0012
MAN2B1	mannosidase alpha class 2B member 1	1144.97	0.62	0.0000	0.0002
MANBA	mannosidase beta	883.39	0.56	0.0000	0.0000
PPT1	palmitoyl-protein thioesterase 1	240.13	0.90	0.0000	0.0000
SCARB2	scavenger receptor class B member 2	5275.19	0.97	0.0000	0.0000
SLC11A2	solute carrier family 11 member 2	637.07	0.55	0.0005	0.0026
SLC17A5	solute carrier family 17 member 5	1176.23	0.48	0.0000	0.0000
SORT1	sortilin 1	1832.34	0.27	0.0003	0.0016
TPP1	tripeptidyl peptidase 1	2157.41	0.56	0.0000	0.0000

[1]**log2FoldChange:** log2 fold change between the groups; [2]**pvalue:** Wald test p-value; [3]**padj:** Benjamini-Hochberg adjusted p-value.

Table S4: Oligonucleotides used for the amplification and sequencing of the 6 exons of the *SMPD1* gene and adjacent intronic regions are shown. All oligonucleotides were designed with the specific sequence of the *SMPD1* gene (genomic *SMPD1* reference sequence NG_011780).

Exon *SMPD1* Gene	Amplicon Size (pb)	Forward Primer (5´-3´)	Reverse Primer (5´-3´)
1	544	agaagggtaatcgggtgtcc	tagatgccaccctctccatc
2	996	tggaaatggaggcccaag	ttaggggagccaaatgaaga
3, 4	667	actgtgagctccttgcaggt	tgctcaagggaattttcagc
5, 6	976	ggggaggctcctcactagaa	agctccaggaaaggagaagg
6	846	agggctcgagaaacctatgg	atgggcacctctgctgtatt
1	544	agaagggtaatcgggtgtcc	tagatgccaccctctccatc

Table S5: Specific primers for QT-PCR amplification of genes LGR5, KRT19, ALB and APOB. TaqMan probes Universal probe library, UPL, (Roche).

Gene Name*	Forward Primer (5´-3´)	Reverse Primer (5´-3´)	Universal Probe Library for Human (Roche)
LGR5	accagactatgcctttggaaac	ttcccagggagtggattctat	#78, cat.no. 04689011001
KRT19	gccactactacacgaccatcc	caaacttggttcggaagtcat	#71, cat.no. 04688945001
ALB	agaggtctcaagaaacctaggaaa	ggttcaggaccacggataga	#44, cat.no. 04688040001
APOB	acagctgattgaggtgtcca	agccactggaggatgtgagt	#90, cat.no. 04689151001

*Gene Name: LGR5 (Leucine-rich repeat containing G protein-coupled receptor 5), KRT19 (Keratin 19), ALB (Albumin) and APOB (Apolipoprotein B).

Table S6: Specific primers of sphigomienlin metabolism genes are shown for quantitative analysis of their expression.

Gene Name	Forward Primer (5´-3´)	Reverse Primer(5´-3´)	Protein Name
SGMS1	ttctggtggtatcacactatg	gtaagatcgaggtacaattcc	Sphingomyelin Synthase 1 (SMS1)
SMPD1	ctcgggctgaagaaggaaccca	agcgtctccacacctccaccat	Acid Sphingomyelinase (ASM)
SMPD2	ctgtttctcaaggtgcag	acgaggaggtagattttcg	Sphingomyelin phosphodiesterase 2 (nSM2)
GAPDH	cctcgtcccgtagacaaaatg	tgaaggggtcgttgatggc	Glyceraldehyde-3-phosphate Dehydrogenase (GAPDH)
